# Cumulative Associations Between Midlife Health Behaviors and Physical Functioning in Early Old Age: A 17-Year Prospective Cohort Study

**DOI:** 10.1111/jgs.13071

**Published:** 2014-10-03

**Authors:** Séverine Sabia, Alexis Elbaz, Nicolas Rouveau, Eric J Brunner, Mika Kivimaki, Archana Singh-Manoux

**Affiliations:** * Department of Epidemiology and Public Health, University College LondonLondon, UK; † INSERM, U1018, Centre for Research in Epidemiology and Population HealthVillejuif, France; ‡ University Paris 11Villejuif, France; § PRES Sorbonne Paris Cité, Université Paris DescartesFrance; Ö Centre de Gérontologie, Hôpital Ste Périne, Assistance Publique-Hôpitaux de ParisParis, France

**Keywords:** health behaviors, walking speed, upper-limb strength, physical function

## Abstract

**Objectives:**

To examine cumulative associations between midlife health behaviors and walking speed and upper-limb strength in early old age.

**Design:**

Prospective cohort study.

**Setting:**

Whitehall II Study.

**Participants:**

Individuals (mean age 49.1 ± 5.9 in 1991–93) with health behavior data for at least two of the three assessments (1991–93, 1997–99, 2002–04) and physical functioning measures in 2007–09 (mean age 65.9 ± 5.9) (N = 5,671).

**Measurements:**

A trained nurse assessed walking speed and upper-limb strength. Unhealthy behaviors were defined as current or recent smoking, nonmoderate alcohol consumption (abstinence or heavy drinking), fruit and vegetable consumption less than twice per day, and physical inactivity (<1 h/wk of moderate and <1 h/wk of vigorous physical activity). For each unhealthy behavior, a cumulative score was calculated as the number of times a person reported the behavior over the three assessments divided by 3. The score ranged between 0 (never) and 1 (all three times).

**Results:**

In linear regression models adjusted for age, sex, education, marital status, and height, all unhealthy behaviors in 1991–93 were associated with slower walking speed in 2007–09, with differences ranging from 0.10 (nonmoderate alcohol consumption) to 0.25 (physical inactivity) of a standard deviation between participants with and without the unhealthy behavior (*P*_*t*-test_<.001). For walking speed, the accumulation-of-risk model provided the best fit for unhealthy diet (*β* for a 1-point increment in the low fruit and vegetable consumption score = −0.29, 95% confidence interval (CI) = −0.36 to −0.22) and physical inactivity (*β *= −0.37, 95% CI = −0.45 to −0.29). For smoking and nonmoderate alcohol consumption, a cumulative effect was also observed, but partial *F*-tests did not suggest that it provided a better fit than models with behaviors in 1991–93, 1997–99, or 2002–04. All behavioral scores except smoking were associated with grip strength, but *F*-tests supported the accumulation-of-risk hypothesis only for physical inactivity.

**Conclusion:**

These findings highlight the importance of duration of unhealthy behaviors, particularly for diet and physical activity, when examining associations with physical functioning.

Objective measures of physical functioning such as walking speed and upper limb strength predict mortality and other adverse health outcomes,^1^–^3^ but the determinants of poor physical function at older ages, particularly modifiable factors, are not well known. Previous studies suggest that health behaviors—smoking,^4^–^7^ alcohol consumption,^6^ diet,^8^–^12^ and physical activity^6^,^13^–^21^—are related to physical function. Plausible underlying mechanisms include vascular disease^22^ and poor cognitive function,^23^,^24^ because health behaviors are associated with these outcomes that in turn are related to physical function.^25^–^29^

Aging is a continuous process, resulting from the accumulation of damage over time,^30^ making it important to consider the duration of exposure to risk factors when examining aging outcomes. Most studies on the association between health behaviors and physical function have assessed them at only one time point,^4^,^6^,^8^–^13^,^15^,^17^–^19^,^21^ although previous studies have found associations between pack-years of smoking^4^–^6^ and physical function, as well as a cumulative effect of physical activity.^7^,^14^,^16^,^20^ Nevertheless, it remains unclear whether there is a cumulative effect from midlife (<65) to early old age for the effect of health behaviors on physical function. Furthermore, although health behaviors are correlated,^31^,^32^ most but not all^4^,^6^–^8^,^10^ studies have focused on single health behaviors rather than consider multiple behaviors simultaneously.^5^,^9^,^11^,^13^–^16^,^18^–^21^

In the present report from the Whitehall II study, repeat data on health behaviors (diet, physical activity, smoking, alcohol consumption) assessed three times over a 12-year period were used to examine whether the accumulation of unhealthy behaviors from midlife to early old age is associated with physical function in early old age. Walking speed and upper limb strength were used to assess physical function at follow-up objectively, and accumulation effects were compared with effects of health behaviors assessed 17, 10, and 5 years before the measure of physical functioning. It was hypothesized that the effect of unhealthy behaviors on function would increase with duration of exposure, in terms of number of times the unhealthy behavior was reported over a 12-year period. Given the clustering of risk factors, it was anticipated that the association between each health behavior and function would be attenuated when other health behaviors are taken into account.

## Methods

### Study Population

Data were drawn from the Whitehall II cohort study, established in 1985–88 and involving 10,308 individuals (67% male) aged 35 to 55.^33^ Participants provided written consent to participate in the study, and the University College London ethics committee approved the study. Study design consists of a clinical examination approximately every 5 years: 1985–88, 1991–93, 1997–99, 2002–04, and 2007–09. For the present study, baseline is defined as 1991–93. The analytical sample consists of the 5,671 participants with physical function measures in 2007–09 and health behaviors assessed in 1991–93, 1997–99, and 2002–04.

### Health Behaviors

Data on health behaviors were assessed using questionnaires three times (1991–93, mean age 49.1 ± 5.9; 1997–99, mean age 55.4 ± 5.9; 2002–04, mean age 60.9 ± 5.9). Data missing at one wave were replaced with those from the wave immediately before or after that wave, including the 1985–88 and 2007–09 waves. Data were imputed once over the three waves for one health behavior for 357 (6.3%) participants, for two health behaviors for 26 (0.5%) participants, for three health behaviors for 65 (1.1%) participants, and for four health behaviors for 519 (9.2%) participants.

Smoking status was assessed using questions on current and past cigarette smoking, categorized as current, ex- or never-smoker. Current and recent ex- (cessation since the previous wave) smoking were defined as unhealthy.^7^,^34^

Alcohol consumption was assessed using questions on the number of alcoholic drinks (measures of spirits, glasses of wine, and pints of beer) consumed in the last 7 days, converted to number of units of alcohol, with each unit corresponding to 8 g of ethanol. Alcohol consumption was categorized as no or occasional (no alcohol in the last week), moderate (1–14 U/wk in women, 1–21 U/wk in men), and heavy (≥14 U/wk in women, ≥21 U/wk in men). Consistent with previous studies, alcohol consumption other than moderate was defined as unhealthy.^34^–^37^

Fruit and vegetable consumption was assessed using the question “How often do you eat fresh fruit or vegetables?” Responses were on an 8-point scale ranging from seldom or never to two or more times a day. Eating fruits and vegetables less than twice daily was defined as having an unhealthy diet.^38^

In 1991–93, participants were asked about frequency and duration of participation in mildly energetic (e.g., weeding, general housework, bicycle repair), moderately energetic (e.g., dancing, cycling, leisurely swimming), and vigorous (e.g., running, hard swimming, playing squash) physical activity. Examples of each level of physical activity were provided to allow similar interpretation of the items by the participants. In subsequent assessments from 1997–99 to 2007–09, the questionnaire included 20 items on frequency and duration of participation in different physical activities (e.g., walking, cycling, sports) that were used to compute hours per week of each intensity level. Participants reporting less than 1 h/wk of moderate and less than 1 h/wk of vigorous physical activity were classified as inactive, the unhealthy behavior.^38^

### Physical Functioning (2007–09)

Walking speed was measured over an 8-foot (2.44-m) marked course.^39^,^40^ Participants were asked to “walk to the other end of the course at [their] usual walking pace, just as if [they] were walking down the street to go the shops.” The starting position was standing at the start of the course. A trained nurse walked behind the participant and stopped timing when the participant's foot hit the floor after the end of the walking course. Three tests were performed, and walking speed (m/s) was computed as the distance divided by the mean of the three trials to complete the test.

Upper limb strength was assessed using a test of grip strength (kg) of the dominant hand using a handgrip dynamometer adjusted to suit participants' hands. Participants were seated with their elbow on the table, the forearm pointing upward, and the palm of the hand facing up. They were asked to squeeze the dynamometer as hard as they could for 2 or 3 seconds. Three tests were conducted with a 1-minute break between each measure, and the maximum of the three tests was used for the analyses.

Covariates included age, sex, height measured at the 2007–09 clinical examination, marital status (married or cohabiting vs other) and education (< primary school (to age 11), lower secondary school (to age 16), higher secondary school (to age 18), university, and higher university degree). Body mass index in 1991–93 was calculated as weight (in kilograms) divided by height (in meters) squared. Weight was measured in underwear to the nearest 0.1 kg on electronic scales with digital readout (Soehnle; Leifheit AS, Nassau, Germany). Height was measured in bare feet to the nearest millimeter using a stadiometer with the participant standing erect with head in the Frankfurt plane. Mobility limitations (any limitations in climbing several flights of stairs or walking >1 mile) assessed in 1991–93 and 1997–99 were used in sensitivity analyses.

### Statistical Analysis

Descriptive analyses were conducted to determine baseline characteristics of participants. For each unhealthy behavior, a score was calculated to represent the number of times a person reported the behavior between 1991–93 and 2002–04 divided by three, with scores ranging from 0 (no unhealthy behavior over this period) to 1 (unhealthy behavior at all three waves (1991–93, 1997–99, 2002–04)). To allow comparability of tests, physical function measures were standardized using a *z*-transformation (mean = 0, standard deviation = 1). Linear regression models were used to examine the association between each behavior and standardized physical function measures in 2007–09. Analyses were conducted for health behaviors in 1991–93 (17 years before the measure of physical functioning), 1997–99 (10 years before), 2002–04 (5 years before), and finally for the cumulative scores of unhealthy behaviors over the three assessments. A structured approach was used to compare the fit of different models.^41^ *F*-statistics were used to compare each model with a saturated model (including the three measures of health behavior over time and their interactions); large *P*-values indicate that a given restricted model is as good as the saturated model in fitting the data. For nonnested models, the Akaike information criterion (AIC) was used to compare the models' fit; a lower AIC indicates better fit. Models were first adjusted for potential confounders (age, sex, height, marital status, education) and then mutually adjusted for all health behaviors and for body mass index (BMI) in 1991–93 because it is strongly associated with health behaviors and physical function. Finally, the association with the number of unhealthy behaviors (ranging from 0 (no unhealthy behavior) to 4 (all unhealthy behaviors)) at each wave (1991–93, 1997–99, 2002–04), as well as with their sum over the 1991–93 to 2002–04 period (ranging from 0 (no unhealthy behaviors at all three waves) to 12 (all unhealthy behaviors at the three waves)) were investigated. *P*-values reported are two-sided. Analyses were performed with SAS version 9.3 (SAS Institute, Inc., Cary, NC).

#### Sensitivity Analyses

Multiple imputation was used to account for missing data on health behaviors during the follow-up period. In further analysis, participants who reported limitations in mobility in 1991–93 or 1997–99 might have modified their health behaviors over the follow-up. To assess for this bias, sensitivity analyses were performed excluding these participants.

## Results

Of the 8,815 participants in the 1991–93 wave of data collection, 730 died before the physical functioning assessment in 2007–09, and 5,998 participated in this assessment (questionnaire, clinical examination, or both), of whom 5,892 had measures of walking speed, grip strength, or both (Figure S1); 5,671 of these participants had health behavior data for at least two of the three assessments (1991–93, 1997–99, 2002–04), constituting the analytical sample. Participants in the analytical sample were younger than the 2,414 participants alive in 2007–09 and excluded from the analysis because of missing data on health behaviors or physical functioning (49.1 vs 50.1 in 1991–93, *P* = .05) and more likely to be male (72.1% vs 61.4%, *P* < .001) and have higher education (30.2% vs 22.9% with university degree or more, *P* < .001). Those included in the analysis were less likely to be smokers (11.4% vs 19.0%), nonmoderate alcohol drinkers (33.9% vs 41.2%), inactive (18.1% vs 25.0%), and eat fruits and vegetable less than twice a day (77.6% vs 81.9%) in 1991–93 (all *P* < .001).

Table 1 shows the characteristics of the study population as a function of cumulative scores, consisting of repeated measures of each unhealthy behavior. At baseline, 14.7% of participants were current or recent ex-smokers, 33.9% did not report moderate alcohol consumption in the previous week (48% abstainers, 52% heavy drinkers), 77.6% consumed fruits and vegetables less than twice daily, and 18.1% were not physically active. Unhealthy behaviors were reported two or three times out of the three waves for 12.3% of participants for current or recent ex-smoking, 35.0% for nonmoderate alcohol consumption, 67.4% for fruit and vegetable consumption less than twice daily, and 20.5% for physical inactivity. The distributions of the cumulative scores of health behaviors are presented in Figure S2. The average walking speed in participants included in the analysis was 1.10 ± 0.27 m/s, and the average grip strength was 37.8 ± 10.6 kg.

**Table 1:** Baseline Characteristics of the Study Population as a Function of Unhealthy Behaviors Between 1991–93 and 2002–04

Baseline Characteristic	Study Population, N = 5,671	Unhealthy Behaviors 2 or 3 Times in the Three Assessments (1991–93, 1997–99, 2002–04)			
		Current or Recent Ex-Smoking, n = 700 (12.3%)	Nonmoderate Alcohol Consumption, n = 1,984 (35.0%)	Fruit and Vegetable Consumption < Twice Daily, n = 3,823 (67.4%)	Physical Inactivity, n = 1,160 (20.5%)
Age, mean ± SD	49.1 (5.9)	48.4 (5.6)^a^	49.0 (5.9)	49.0 (5.9)^a^	48.6 (5.9)^a^
Male, n (%)	4,086 (72.1)	472 (67.4)^a^	1,342 (67.6)^a^	2,882 (75.4)^a^	649 (56.0)^a^
Height, cm, mean ± SD (in 2007–09)	171.0 (9.2)	170.1 (9.3)^a^	170.3 (9.5)^a^	171.2 (9.2)	167.9 (10.1)^a^
Married or cohabiting, n (%)	4,354 (76.8)	496 (71.0)^a^	1,436 (72.4)^a^	2,915 (76.3)	748 (64.5)^a^
≥University degree, n (%)	1,715 (30.2)	141 (20.1)^a^	601 (30.3)	1,007 (26.3)^a^	333 (28.7)
Current or recent ex-smoking, n (%)	834 (14.7)	669 (95.6)^a^	376 (19.0)^a^	660 (17.3)^a^	211 (18.2)^a^
Nonmoderate alcohol consumption, n (%)	1,922 (33.9)	298 (42.6)^a^	1,538 (77.5)^a^	1,349 (35.3)^a^	491 (42.3)^a^
Fruit and vegetable consumption < twice daily, n (%)	4,403 (77.6)	623 (89.0)^a^	1,563 (78.8)	3,683 (96.3)^a^	947 (81.6)^a^
Physical inactivity, n (%)	1,025 (18.1)	158 (22.6)^a^	419 (21.1)^a^	741 (19.4)^a^	691 (59.6)^a^

Unhealthy behaviors were defined as current and recent ex- (cessation since the previous wave) smoking, nonmoderate alcohol consumption (abstinence or alcohol consumption ≥14 U/wk in women, ≥21 U/wk in men), fruit and vegetable consumption < twice daily, and physical inactivity (<1 h/wk of moderate and <1 h/wk of vigorous physical activity).

a *P* <.05 for the difference in participant characteristics according to cumulative score for each unhealthy behavior (2 or 3 times vs never or once).

SD = standard deviation.

Table 2 shows the association between each unhealthy behavior in 1991–93, 1997–99, and 2002–04 and cumulative scores over time and walking speed in 2007–09. In models adjusted for sociodemographic characteristics, all unhealthy behaviors were associated with slower walking speed, regardless of wave of assessment. Higher cumulative behavioral scores over time were associated with slower walking speed (Figure S3, Table 2). Partial *F*-test and AIC suggested that the accumulation-of-risk models fit the data better than the models with one assessment of unhealthy behaviors for unhealthy diet and physical inactivity (*P* ≥ .15 for partial *F*-test for the cumulative score and <.005 for the measures of diet and physical activity at different time points (1991–93, 1997–99, 2002–04)). Betas corresponding to difference in standardized walking speed between participants who never reported the unhealthy behavior and those who reported it at all three waves, assuming linearity in associations, were −0.29 (95% confidence interval (CI) = −0.36 to −0.22) for unhealthy diet and −0.37 (95% CI = −0.45 to −0.29) for physical inactivity. For smoking and nonmoderate alcohol consumption, the tests did not permit to distinguish between the models. Smoking in 1991–93 and 1997–99 and cumulative score all fit the data better than a saturated model (*P* > .05 for partial *F*-test); the AIC was similar for the smoking measure in 1997–99 and the cumulative score. For alcohol consumption, the accumulation-of-risk model and the model using alcohol consumption in 1991–93 (*P*-values for partial *F*-test >.05) fit the data better than the models including alcohol consumption in 1997–99 or 2002–04 (*P* < .005 for partial *F*-test).

**Table 2:** Association Between Unhealthy Behaviors Between 1991–93 and 2002–04 and Standardized Scores of Walking Speed in 2007–09 (N = 5,622)

Behavior	Association with Unhealthy Behaviors		Partial *F*-Test Against Saturated Model^a^		Akaike Information Criterion
	*β*^b^	95% Confidence Interval	*F*-Statistic	*P*-Value	
Smoking					
1991–93 (17 years before)	−0.18	−0.25 to −0.11	1.50	.19	−788.83
1997–99 (10 years before)	−0.22	−0.29 to −0.14	0.57	.72	−793.46
2002–04 (5 years before)	−0.20	−0.28 to −0.12	2.30	.04	−784.85
Accumulation of risk^c^	−0.23	−0.31 to −0.15	0.61	.69	−793.27
Nonmoderate alcohol consumption					
1991–93 (17 years before)	−0.13	−0.18 to −0.08	0.47	.83	−786.903
1997–99 (10 years before)	−0.08	−0.13 to −0.03	2.97	.007	−771.898
2002–04 (5 years before)	−0.07	−0.12 to −0.02	3.46	.002	−768.997
Accumulation of risk^c^	−0.15	−0.21 to −0.08	1.20	.30	−782.546
Fruit and vegetable consumption < twice daily					
1991–93 (17 years before)	−0.14	−0.20 to −0.08	9.83	<.001	−784.019
1997–99 (10 years before)	−0.19	−0.24 to −0.14	4.54	<.001	−815.575
2002–04 (5 years before)	−0.20	−0.24 to −0.15	3.54	.002	−821.545
Accumulation of risk^c^	−0.29	−0.36 to −0.22	1.56	.15	−833.454
Physical inactivity					
1991–93 (17 years before)	−0.23	−0.30 to −0.17	6.14	<.001	−810.990
1997–99 (10 years before)	−0.18	−0.24 to −0.12	7.58	<.001	−802.354
2002–04 (5 years before)	−0.16	−0.22 to −0.10	9.27	<.001	−792.280
Accumulation of risk^c^	−0.37	−0.45 to −0.29	1.44	.19	−839.103

a Comparing the fit of the corresponding model against a saturated model including unhealthy behavior variables at all three measurement periods and their interactions.

b Mean difference in standardized walking speed. Models are adjusted for age, sex, educational level, marital status, and height.

c Estimates are for a 1-point increment in the cumulative score of the unhealthy behavior under consideration assuming a linear association between the number of times a person was classified as having the unhealthy behavior in the three assessments (1991–93, 1997–99, 2002–04) and walking speed.

In addition to smoking, there was some evidence of accumulation of risk over time with grip strength as the outcome, but the associations were generally weaker than those with walking speed (Table 3, Figure S3). The exception was the association with physical inactivity, for which the cumulative association score provided the best fit to the data (*β *= −0.38; 95% CI = −0.44 to −0.32; *P* > .05 for partial *F*-test and lowest AIC of the models including physical activity at one time point).

**Table 3:** Association Between Unhealthy Behaviors over 1991–93 to 2007–09 with Standardized Scores of Grip Strength in 2007–09 (N = 5,602)

Behavior	Association with Health Behaviors		Partial *F*-Test Against Saturated Model^a^		Akaike Information Criterion
	*β*^b^	95% Confidence Interval	*F*-Statistic	*P*-Value	
Smoking					
1991–93 (17 years before)	−0.01	−0.06–0.04	0.81	.54	−4,303.70
1997–99 (10 years before)	−0.02	−0.08–0.04	0.77	.57	−4,303.93
2002–04 (5 years before)	−0.03	−0.09–0.03	0.73	.60	−4,304.13
Accumulation of risk^c^	−0.02	−0.04–0.03	0.76	.58	−4,303.96
Nonmoderate alcohol consumption					
1991–93 (17 years before)	−0.07	−0.11 to −0.03	0.70	.65	−4,317.34
1997–99 (10 years before)	−0.01	−0.04–0.03	3.00	.006	−4,303.56
2002–04 (5 years before)	−0.02	−0.06–0.01	2.80	.01	−4,304.79
Accumulation of risk^c^	−0.05	−0.10 to −0.01	2.23	.04	−4,308.20
Fruit and vegetable consumption < twice daily					
1991–93 (17 years before)	−0.04	−0.08–0.01	1.33	.24	−4,306.91
1997–99 (10 years before)	−0.05	−0.08 to −0.01	0.98	.44	−4,309.04
2002–04 (5 years before)	−0.06	−0.10 to −0.02	0.28	.95	−4,313.20
Accumulation of risk^c^	−0.08	−0.13 to −0.03	0.27	.95	−4,313.30
Physical inactivity					
1991–93 (17 years before)	−0.14	−0.18 to −0.09	22.03	<.001	−4,334.43
1997–99 (10 years before)	−0.19	−0.23 to −0.15	12.80	<.001	−4,388.90
2002–04 (5 years before)	−0.23	−0.27 to −0.19	7.13	<.001	−4,422.67
Accumulation of risk^c^	−0.38	−0.44 to −0.32	2.05	.06	−4,453.08

a Comparing the fit of the corresponding model against a saturated model including unhealthy behavior variables at all three measurement periods and their interactions.

b Mean difference in standardized grip strength. Models are adjusted for age, sex, educational level, marital status, and height.

c Estimates are for a 1-point increment in the cumulative score of the unhealthy behavior under consideration assuming a linear association between the number of times a person was classified as having the unhealthy behavior in the three assessments (1991–93, 1997–99, 2002–04) and grip strength.

**Figure 1:**
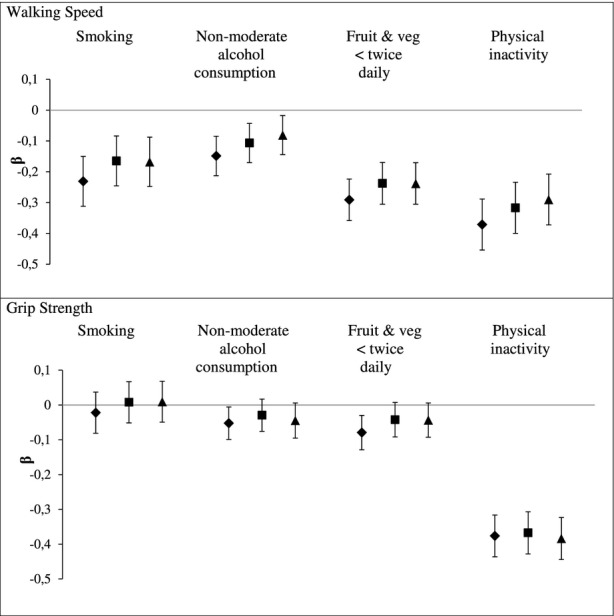
Cumulative effect of unhealthy behaviors (1991–93 to 2002–04) on physical functioning in 2007–09 before and after mutual adjustment for health behaviors, and additionally adjusted for body mass index (BMI). *β* represents mean difference in standardized score of physical functioning. Models are adjusted for age, sex, educational level, marital status, and height (and mutually adjusted for health behavior scores for bold square results). Estimates are for a 1-point increment in cumulative score of the unhealthy behavior under consideration assuming a linear association between the number of times a person was classified as having the unhealthy behavior in the three assessments (1991–93, 1997–99, and 2002–04) and physical functioning. ♦: Each health behavior separately; ■: Health behaviors mutually adjusted; ▲: Additionally adjusted for BMI.

Figure 1 presents the cumulative associations between the unhealthy behaviors and physical function before and after mutual adjustment for all health behavior scores. For walking speed, associations were attenuated after adjustment by 15% to 34%, depending on the behavior in question (*β* after adjustment = −0.16 for smoking, −0.11 for nonmoderate alcohol consumption, −0.24 for low fruit and vegetable consumption, and −0.32 for physical inactivity). For grip strength, the associations with nonmoderate alcohol consumption and low fruit and vegetable consumption were no longer statistically significant (44% and 47% reduction, respectively), whereas the association with physical inactivity was attenuated by only 2% (*β *= −0.37). Participants with higher BMI in 1991–93 were more likely to have an unhealthy diet, to be nonmoderate alcohol drinkers, and physical inactive, and to have lower walking speed and greater grip strength (all *P* < .001). Additional adjustment for BMI slightly attenuated the associations between cumulative scores of health behaviors and walking speed but did not influence the association between physical inactivity and grip strength.

**Figure 2:**
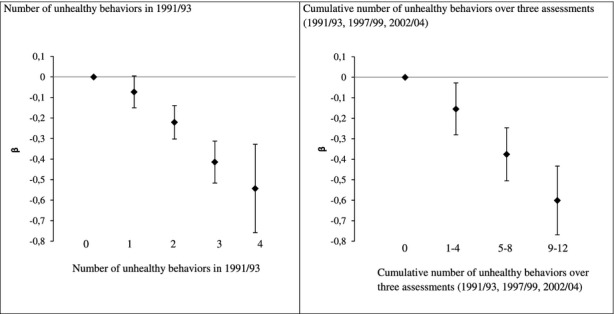
Association of the number of unhealthy behaviors in 1991/93 and between 1991/93 and 2002/04 with walking speed in 2007/09.

Because all unhealthy behaviors were independently associated with walking speed, associations with the number of unhealthy behaviors in 1991–93 and the cumulative number of unhealthy behaviors over the three time points were assessed (Figure 2). A higher number of unhealthy behaviors in 1991–93 was associated with slower walking speed at the end of follow-up (*β* comparing those with four unhealthy behaviors with those with no unhealthy behaviors in 1991–93 = −0.53, 95% CI = −0.77 to −0.30). A better fit with the data was observed for the cumulative number of unhealthy behaviors over the 1991–93 to 2002–04 period than for the model including the number of unhealthy behaviors at one time point (*P*_partial *F*-test_ <.001 comparing each model including the number of unhealthy behaviors in 1991–93, 1997–99, or 2002–04 with the saturated model, and *P*_partial *F*-test_ = .45 comparing the model including the cumulative score with the saturated model). This analysis was not repeated for grip strength because only physical inactivity was associated with grip strength in analyses adjusted for other unhealthy behaviors (Figure 1).

### Sensitivity Analyses

When using multiple imputation to account for missing data on health behaviors over follow-up, results remained comparable with those presented in main analysis (**Table S1). In further analyses, participants who reported mobility limitations in 1991–93 were excluded from the analysis. This analysis was then repeated excluding those who reported mobility limitations in 1991–93 or 1997–99. The results no longer showed an association between nonmoderate alcohol consumption and walking speed and showed a less-pronounced association with physical inactivity (Table S2). Results were unchanged for grip strength.

## Discussion

This report, based on three measures of unhealthy behaviors over 12 years and objective measures of walking speed and upper limb strength 5 years after the last assessment of health behaviors, presents four important findings. One, all midlife unhealthy behaviors examined (smoking, nonmoderate alcohol consumption, low fruit and vegetable consumption, physical inactivity) were associated with slower walking speed, a measure of function that involves the whole body, 17 years later. Nonmoderate alcohol consumption and physical inactivity were associated with upper limb strength, assessed using grip strength, at the end of follow-up. Two, there was evidence of accumulation of risk from low fruit and vegetable consumption and physical inactivity for walking speed. For smoking and nonmoderate alcohol consumption, although an association was found with cumulative scores, there was no clear evidence that the accumulation-of-risk hypothesis provided the best fit to the data. For grip strength, there was evidence of accumulation of risk from physical inactivity. Three, mutual adjustment for health behaviors attenuated the associations. Four, greater cumulative number of unhealthy behaviors over midlife was associated with slower walking speed in early old age.

Most previous studies have examined the effect of health behaviors based on measurements at one time point;^4^,^6^,^8^–^13^,^15^,^17^–^19^,^21^ the cumulative effect of health behaviors over time has been explored to a lesser extent.^4^–^6^,^14^,^16^,^20^ Some previous studies showed a cumulative effect of smoking, using pack-years of smoking, on a composite score of physical function^6^ and on lower body physical function^4^,^5^ but not on upper limb strength.^5^ At least three studies have investigated the cumulative effect of physical activity. One retrospective study found a higher cumulative physical activity score in midlife to be associated with better mobility in older age.^16^ Another study reported a cumulative effect of midlife physical activity on lower body function but not on grip strength at age 53.^14^ Finally, a study based on 229 older women found a cumulative effect of physical activity on walking speed.^20^ In the present study, to the authors' knowledge for the first time, whether a cumulative score over time provided a better fit to the data than a measure of unhealthy behavior at one time point was assessed.^41^ The results suggest accumulation of risk for the association between low fruit and vegetable consumption and walking speed and for the association between physical inactivity and both walking speed and upper limb strength. For smoking and nonmoderate alcohol consumption, accumulation-of-risk models and models including measures at one time point fit equally well with the data. It is possible that, for these behaviors, and particularly for smoking, the time elapsed between the measurements was not long enough to allow the detection of accumulation of risk.

Unhealthy behaviors are known to cluster in individuals.^31^,^32^ Despite this finding, most studies have focused on single health behaviors without taking into account the effect of other behaviors.^5^,^9^,^11^,^13^–^16^,^18^–^21^ The few exceptions include a study showing that alcohol consumption less than five times in the previous year, exercising less than three times a week, and having smoked more than 100 cigarettes in a lifetime were all independently associated with lower physical functioning.^6^ Two others studies found smoking, but not physical activity and heavy alcohol use, to predict decline in lower body physical function^4^ and handgrip strength,^7^ and two further studies reported an association between Mediterranean diet and physical function in analyses taking into account smoking and physical activity,^8^,^10^ although another study found that physical activity largely explained the association between the Healthy Eating Index and physical function.^12^ The current study adds to this evidence by showing that the association between a health behavior and physical function was attenuated by 2% to 47% after adjustment for other behaviors, depending on the health behavior and the test of physical function under consideration. There was no longer a cumulative association between nonmoderate alcohol consumption and fruit and vegetable consumption and grip strength after taking other health behaviors into account. Furthermore, all unhealthy behaviors were independently associated with walking speed, and participants reporting more unhealthy behaviors over time were found to walk more slowly.

Physical inactivity was found to be associated with walking speed and upper limb strength. Physical activity increases muscle strength and improves balance, both of which are associated with better physical functioning,^42^,^43^ although because there was only one assessment of motor function at the end of follow-up, it was not possible to examine decline in motor function. Thus, it cannot be excluded that participants with poorer physical performance also had poor physical function at baseline, which may have influenced physical activity. To address this issue, sensitivity analysis excluding participants who reported mobility limitations at any of the first two waves were performed and showed an association between physical inactivity score and upper limb strength similar to that in the main analysis, although the association with walking speed was 53% lower, suggesting that reverse causation could explain part of the observed association, in that changes in physical function that occurred earlier in life might have influenced physical activity over the follow-up. Additional longitudinal research is needed to estimate the extent to which physical limitations present early in adulthood confound the association between physical activity and physical function.

Multiple mechanisms are likely to underlie the association between health behaviors and physical function. The vascular pathway is likely to be important because unhealthy behaviors contribute to the development of several vascular outcomes^22^,^44^ related to poorer physical function.^25^,^27^,^45^–^47^ Health behaviors are also associated with cognitive outcomes,^23^,^24^ and there is considerable research showing associations between cognitive and physical function.^26^,^28^,^29^ Further plausible mechanisms may involve the musculoskeletal^48^ and pulmonary systems.^40^

The current study has some limitations. First, because the findings are from an occupational cohort, the participants are likely to be healthier than the general population. Second, during the 17-year follow-up, the 29.9% of the target population that was lost to follow-up tended to have more unhealthy behaviors at baseline. Because participants who dropped out were also more likely to have health problems,^49^ the associations reported here might have been underestimated. This might also contribute to the lack of support for the accumulation-of-risk hypothesis in relation to smoking. There were fewer smokers in the study sample than in excluded participants. Fourth, data on health behaviors were assessed using self-report and were thus subject to potential measurement error. Fifth, health behaviors were analyzed as binary variables to facilitate interpretation of the cumulative scores. This might have resulted in better characterization of some health behaviors than others; caution is thus required when comparing the effect of different health behaviors. Finally, physical function was measured only once, so it was not possible to examine the effect of health behaviors on change in physical functioning.

In conclusion, this study found an association between midlife health behaviors and physical function in early old age. The results highlight the importance of duration of adverse health behaviors, particularly for diet and physical activity, and their coexistence when examining their association with physical functioning.

## Supporting Information

Table S1 Association between the cumulative score of unhealthy behaviors with standardized scores of physical functioning in 2007/09: analyses based on multiple imputation. **Table S2.** Association between the cumulative score of unhealthy behaviors with standardized scores of physical functioning in 2007/09 among participants who did not report mobility limitations in 1991/93 and 1997/99. **Figure S1.** Flowchart of the study. **Figure S2.** Distribution of the number of unhealthy behaviors between 1991/93 and 2002/04. **Figure S3.** Association of the cumulative score of unhealthy behaviors (1991/93 to 2002/04) with standardized score of physical functioning in 2007/09.
