# Supplementary material for: Cumulative Associations Between Midlife Health Behaviors and Physical Functioning in Early Old Age: A 17-Year Prospective Cohort Study
Source: J Am Geriatr Soc. 2014 Oct 3;62(10):1860–8. doi: 10.1111/jgs.13071 (PMC4206608; doi:10.1111/jgs.13071)
Supplement: Table S1 — Association between the cumulative score of unhealthy behaviors with standardized scores of physical functioning in 2007/09: analyses based on multiple imputation. Table S2. Association between the cumulative score of unhealthy behaviors with standardized scores of physical functioning in 2007/09 among participants who did not report mobility limitations in 1991/93 and 1997/99. Figure S1. Flowchart of the study. Figure S2. Distribution of the number of unhealthy behaviors between 1991/93 and 2002/04. Figure S3. Association of the cumulative score of unhealthy behaviors (1991/93 to 2002/04) with standardized score of physical functioning in 2007/09. [file jgs0062-1860-SD1.docx]

**SUPPLEMENTAL MATERIAL FOR ONLINE ONLY**

**Appendix**

Table S1: Association between the Cumulative Score of Unhealthy Behaviors with Standardized Scores of Physical Functioning in 2007/09: Analyses based on Multiple Imputation.

|  | **WALKING SPEED** | | **GRIP STRENGTH** | |
| --- | --- | --- | --- | --- |
| **Heath behaviors score** | **β**^a^ | **95% CI** | **β**^a^ | **95% CI** |
| **Smoking** | -0.13 | -0.22, -0.05 | 0.04 | -0.02, 0.10 |
| **Non-moderate alcohol consumption** | -0.11 | -0.17, -0.04 | -0.03 | -0.08, 0.02 |
| **Fruit and vegetable consumption < twice daily** | -0.22 | -0.29, -0.15 | -0.02 | -0.08, 0.03 |
| **Physical inactivity** | -0.30 | -0.39, -0.21 | -0.36 | -0.43, -0.30 |

^a^β represents mean difference in standardized physical functioning scores. Models are adjusted for age, sex, educational level, marital status, height and mutually adjusted for health behaviors. Estimates are for 1-point increment in the cumulative score of the unhealthy behavior under consideration assuming a linear association between the number of times a person was classified as having an unhealthy behavior out of the 3 assessments (1991/93, 1997/99, and 2002/04) and physical functioning.

Table S2: Association between the Cumulative Score of Unhealthy Behaviors with Standardized Scores of Physical Functioning in 2007/09 among Participants who Did Not Report Mobility Limitations in 1991/93 and 1997/99

|  | **WALKING SPEED** | | | **GRIP STRENGTH** | | |
| --- | --- | --- | --- | --- | --- | --- |
| **Participants reporting Mobility Limitations in 1991/99 excluded** | **N=3913** | | | | **N=3906** | |
| **Heath behaviors score** | | **β**^a^ | **95% CI** | | **β**^a^ | **95% CI** |
| **Smoking** | | -0.22 | -0.32, -0.11 | | -0.02 | -0.09, 0.06 |
| **Non-moderate alcohol consumption** | | -0.03 | -0.11, 0.05 | | 0.00 | -0.05, 0.06 |
| **Fruit and vegetable consumption < twice daily** | | -0.21 | -0.29, -0.13 | | -0.02 | -0.08, 0.04 |
| **Physical inactivity** | | -0.23 | -0.34, -0.13 | | -0.39 | -0.47, -0.32 |
| **Participants reporting Mobility Limitations in 1991/99 or 1997/99 excluded** | | **N=2979** | | | **N=2971** | |
| **Heath behaviors score** | | **β**^a^ | **95% CI** | | **β**^a^ | **95% CI** |
| **Smoking** | | -0.25 | -0.38, -0.12 | | 0.00 | -0.09, 0.10 |
| **Non-moderate alcohol consumption** | | -0.02 | -0.11, 0.07 | | 0.03 | -0.03, 0.10 |
| **Fruit and vegetable consumption < twice daily** | | -0.21 | -0.30, -0.12 | | -0.02 | -0.09, 0.05 |
| **Physical inactivity** | | -0.15 | -0.27, -0.02 | | -0.41 | -0.51, -0.32 |

^a^β represents mean difference in standardized physical functioning scores. Models are adjusted for age, sex, educational level, marital status, height and mutually adjusted for health behaviors. Estimates are for 1-point increment in the cumulative score of the unhealthy behavior under consideration assuming a linear association between the number of times a person was classified as having an unhealthy behavior out of the 3 assessments (1991/93, 1997/99, and 2002/04) and physical functioning.

Figure S1. Flow-chart of the study

8815 participantsin the 1991/93wave

8085 participants alive in 2007/09

5998 participants undertook clinical examination in 2007/09

5892 participants with walking speed or grip strength measures

5671 participants included in the analyses

730 participants died before the physical functioning assessment in 2007/09

2087 participants did not take part to the clinical examinationsin 2007/09

106 participants did not participate to the physical functioning tests

221 participants with missing data on health behaviors more than once out of the three assessments (1991/93, 1997/99, 2002/04)

Figure S2: Distribution of the Number of Unhealthy Behaviors Between 1991/93 and 2002/04

Smoking Non-moderate Fruit & veg Physical

alcohol< twice inactivity

consumption daily

01 2 3 01 2 3 01 2 3 01 2 3

Number of unhealthy behaviorsover three assessments

(1991/93, 1997/99, 2002/04)

Figure S3: Association of the Cumulative Score of Unhealthy Behaviors (1991/93 to 2002/04) with Standardized Score of Physical Functioning in 2007/09

| Walking speed  0 1 2 3 0 1 2 3 0 1 2 3 0 1 2 3  **Number of unhealthy behaviors over three assessments (1991/93, 1997/99, 2002/04)**  SmokingNon-moderate Fruit & veg Physical  alcohol< twice inactivity  consumptiondaily |
| --- |
| Grip strength  SmokingNon-moderate Fruit & veg Physical  alcohol< twice inactivity  consumptiondaily  0 1 2 3 0 1 2 3 0 1 2 3 0 1 2 3  **Number of unhealthy behaviors over three assessments (1991/93, 1997/99, 2002/04)** |

Footnotes: the cumulative score of unhealthy behavior represents the number of times a person was classified as having the unhealthy behavior under consideration over the 3 assessments (1991/93, 1997/99, 2002/04). For example, for physical inactivity, a score of 0 corresponds to “never” physically inactive over the 3 assessments and a score of 3 to “always” physically inactive over the 3 assessments.
